# Annotations

**Published:** 1887-06-25

**Authors:** 


### ANNOTATIONS.

Hospitals "sitting on the Fence."

OURS is a difficult world to live in. Men are constantly called upon to make decisions between what sometimes are and sometimes only appear to be divergent lines of action. This is so universal in human experience that a variety of proverbs bearing on the subject are in all men's mouths. "No man can serve two masters." "You cannot both run with the hare and hunt with the hounds." "A man cannot both eat his cake and have it." The number might be greatly multiplied, but these are sufficient to show that according to the general judgment of mankind it is sometimes necessary to make a positive and definite choice between two courses, each possessing certain advantages, and the choosing of one of which may lead to the loss of some of the real or supposed advantages of the other. But no man can expect to have all the plums which grow on life's tree, and no hospital can claim to have all sweets and no bitters. Hospitals must be fair and just; like men and women they must learn to "live and let live." Certain hospitals, which Mr. John Biddulph Martin, treasurer of Charing Cross Hospital, undertakes to defend, do not seem to have any faith in these elementary principles, which prevail and must prevail alike in savage and in civilised society, in the nursery and in the cricket-field. These hospitals declare, and, *mirabile dictu*, are not ashamed to declare, that they are willing to sympathise with the Hospital Sunday Fund to the extent of receiving a full proportion of all it has to give them; but when it comes to joining with other hospitals in order to promote the growth of the Fund, then they must pause, lest in their zeal for the Sunday Fund they do anything to check the flow of subscriptions towards their own particular institutions. They are like soldiers who are willing to share the spoils of a campaign, but refuse to march to the battle-field for fear their crops at home should suffer. But, gentlemen, you should make a choice. There is no desire on the part of the Hospital Sunday committee to cut you off from sharing in the spoils; but do you not think honour demands that you should cut yourselves off, seeing that you decline to march to battle on the plea that your marching might injure your prospects at home? Might not every other hospital urge the very same plea, and what then would become of the Sunday Fund, with all its power of educating and arousing the enthusiasm of the public? We are bound to tell you that this sitting upon the fence: this running with the hare and hunting with the hounds, is thoroughly unmanly and un-English, if indeed it be not more appropriately described as "mean." Before the next Sunday Fund collection is made there will be time for reconsideration, and we earnestly hope that then we shall not see three or four, or even one, hospital "sitting upon the fence."

A Happy Thought.

We suggested last week that the Hospital Saturday people should enlist the services of gentlemen as well as ladies for the management of their stalls next year. Whilst we were making the suggestion Liverpool was trying the experiment, and the result has been eminently satisfactory. The weather was fine, the crowds great, the "beggars" sturdy and energetic, and the collection "unusually good." Liverpool is now laying claim to having originated the Saturday Fund. Whether the claim can be established or not is a matter of small importance. It is clear that the public consider it an honourable distinction for any town. The real origin of the idea seems to have been of Dresden inception, and the occasion the necessities of the sick and wounded in the Franco-German war. At first the lady sat patiently by her money-box, and moved only to acknowledge with bow or smile the contributions of the passers-by. In this respect the Dresden model was accurately copied at Liverpool in former years. Latterly, however, the fair pleaders have become more bold, and have impressed into the service of the Fund a number of aides-de-camp in the shape of energetic and irresistible young gentlemen, who, box in hand, perambulate in the region of the stall, and with loud rattle remind the hurrying pedestrian that this is Hospital Saturday, and that "mony a little mak's a muckle," according to the Scotch proverb. Seeing that the experiment has succeeded so well on the banks of the Mersey, it may be desirable for the London fund next year to go and do likewise.

Provident Dispensaries.

During the present year of Jubilee the hospitals are monopolising public attention to the almost total exclusion of the provident dispensaries. Few people will accuse us of deficient regard for the interests of hospitals, or the want of a firm grasp of the voluntary principle as applied to them; but it is incumbent upon us and upon all men to recognise that no one should receive charitable medical aid if he have the means of self-help. Provident dispensaries, if rightly managed, should supply the poor with adequate medical aid on all ordinary occasions. They should deal with every trifling case, leaving only the more serious diseases for treatment at the general hospitals. If this were carried out to any considerable extent, there would be a large and immediate diminution of expenditure in hospital administration. That it is not so carried out is proof of a great want of statesman-like capacity, first on the part of the medical profession, and secondly on the part of hospital managers, who are so overburdened by the demands made upon them. It is abundantly clear that the subject of provident dispensaries must be taken up afresh by men of capacity and courage. It is entirely discreditable to a practical people like ourselves that the two systems of provident and gratuitous medical aid should be in such a condition of hopeless jumble and general inefficiency.

